# Accelerated Course of Alveolar Echinococcosis After Treatment With Steroids in a Patient With Autoimmune Encephalitis

**DOI:** 10.7759/cureus.18831

**Published:** 2021-10-16

**Authors:** Stefan Diem, Bruno Gottstein, Guido Beldi, Nasser Semmo, Lara F Diem

**Affiliations:** 1 Department of Visceral Surgery and Medicine, Bern University Hospital, University of Bern, Bern, CHE; 2 Institute of Infectious Disease, Faculty of Medicine, University of Bern, Bern, CHE; 3 Department of Neurology, Bern University Hospital, University of Bern, Bern, CHE

**Keywords:** case report, liver metastases, reactivation, steroids, immunosuppression, echinococcosis

## Abstract

Human alveolar echinococcosis (AE) is a zoonotic infection caused by the fox tapeworm *Echinococcus *​​​​​​*multilocularis. *We report the case of a patient who developed an accelerated course of AE with diffuse liver involvement after high-dose steroid treatment for autoimmune encephalitis. Immunosuppressive therapies present us with new challenges regarding the management of AE. With this article, we would like to draw attention to the importance of a screening program for AE before planned immunosuppressive therapy.

## Introduction

Human alveolar echinococcosis (AE) is a zoonotic infection caused by the larval form (metacestode) of the fox tapeworm *Echinococcus multilocularis* [1]. For decades before 2000, only sporadic AE infections were reported with Switzerland recording about eight to 12 new AE cases per year [2]. In the last 10-20 years, however, there has been a geographical and numerical increase or expansion within the European endemic area [3]. Switzerland has also been affected by this increase, with now approximately 24-30 new AE cases being annually diagnosed, which corresponds to a two- to three-fold increase compared to the past [2].

Several important factors have been identified as reasons for the expansion of the European endemic area as well as for the increase in AE case numbers in humans. The fox population and concurrently the parasite population itself have increased, on the one hand, due to the successful control of fox rabies, and due to the urbanization of foxes on the other hand. Dogs also act as the definitive host; the parasite was introduced into new areas via this animal species ("traveling" dogs and dog translocations). Another reason is the increased administration of immunomodulatory therapies in humans, with the effect that AE is occurring more frequently as an opportunistic infection. Finally, an overall increased application of crossectional imaging (computer tomography (CT), magnetic resonance tomography (MRI)) is resulting in an increased coincidental early detection of, in most cases, yet asymptomatic AE [4,5].

Vuitton and Gottstein have suggested that immunocompromised (ICR) individuals may present with rapid disease progression compared with immunocompetent patients who seem to be able to control the disease development better, and present with a slowly growing tumor clinically presenting only many years after initial infection [5,6].

We report a case of a male patient who developed an accelerated course of a liver AE, 5.5 months after high dose treatment with steroids (90mg/day (corresponding to 1mg/kgbw/day), initially 1000mg/day intravenously for five days) for autoimmune encephalitis.

## Case presentation

A 59-year-old male with no previous medical or psychiatric history was brought to the hospital following a change of character with apathy, listlessness and fatigue, as well as a deterioration of his general condition and apraxia, which occurred after initial flu-like symptoms and were accompanied by an additional unclear weight loss and a slightly increased C-reactive protein (CRP) value. In addition, in the week prior to admission, there had been repeated transient tingling paresthesias of the left arm, partly accompanied by dysarthria, unilateral facial nerve paralysis, and left arm as well as leg weakness.

The findings in the clinical examination showed neuropsychological deficits with leading frontal brain performance conspicuous with a Montreal Cognitive Assessment (MoCA) test score of 21 out of 30 points. The detailed neuropsychological examination showed a mild to moderate cognitive brain dysfunction (memory, attention, cognitive processing speed, executive functions). The suspicion of the patient's complaints was an autoimmune encephalitis.

On the one hand, this diagnosis was supported by the clinical picture with progressive, subacute neuropsychological and neurocognitive changes. On the other hand, the recurrent transient focal neurological deficits were compatible with focal epileptic seizures [7].

Furthermore, the inflammatory cerebrospinal fluid (CSF) syndrome with a moderate mononuclear pleocytosis (cell count 120 M/L) and negative smear and culture matched autoimmune encephalitis. The positive antibody test in the blood and the CSF for the Ma-2 antibody finally confirmed the diagnosis of a Ma-2 antibody-associated autoimmune encephalitis.

However, the etiology of Ma-2 limbic encephalitis ultimately had to be left open. Since a paraneoplastic genesis occurs in 90% of cases, a broad tumor search including whole-body positron emission tomography (PET) scan was performed, which showed no evidence of a neoplastic event and especially no enhancement of the liver. This was then continued by CT thorax/abdomen every four months.

Therapeutically, we established a seizure-suppressing therapy with oxcarbazepine. Under this therapy, no new epileptic seizures occurred. In addition, in the case of positive antibodies and no evidence of neoplasia, therapy with steroids (1000mg intravenous) was given for five days. This resulted in an improvement in the clinical status with a new score of 26 points on the MoCA test. To avoid the risk of recurrence, a longer-term, immunomodulatory, steroid-sparing therapy with immunoglobulins was started, and the therapy with peroral steroids (initially 1mg/kgbw/day; 90mg/day) was gradually reduced and stopped after four months.

**Figure 1:**
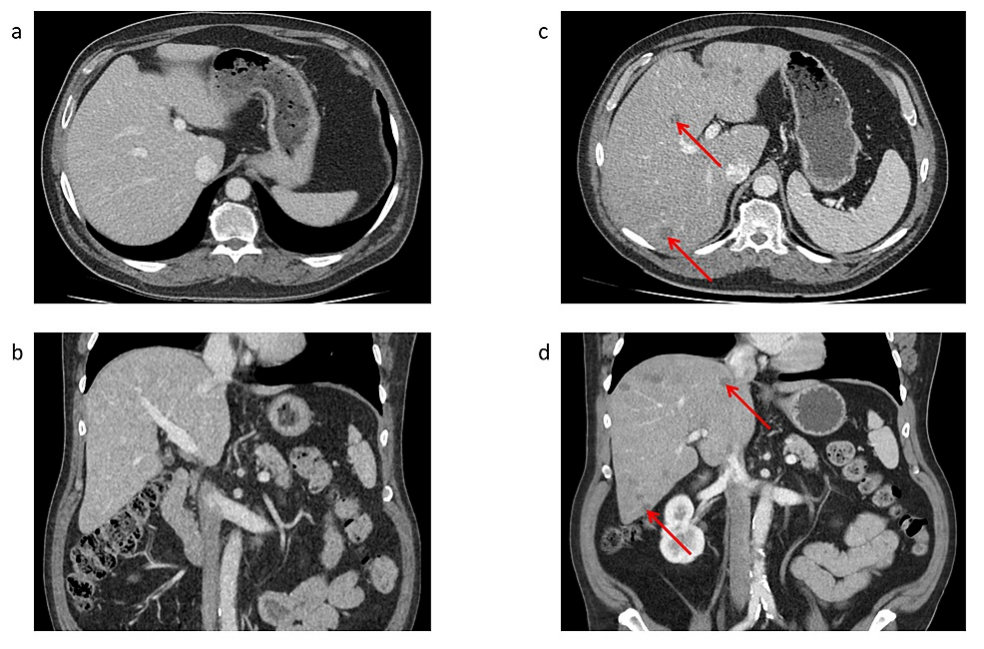
Contrast-enhanced abdominal CT scan Contrast-enhanced abdominal CT scan, (a, b) at time of the diagnosis of autoimmune encephalitis, which reveals normal liver parenchyma, and (c, d) under therapy with glucocorticoids, which reveals multiple hypodense lesions of the liver (arrows). Abbreviations: CT = computer tomography

**Figure 2:**
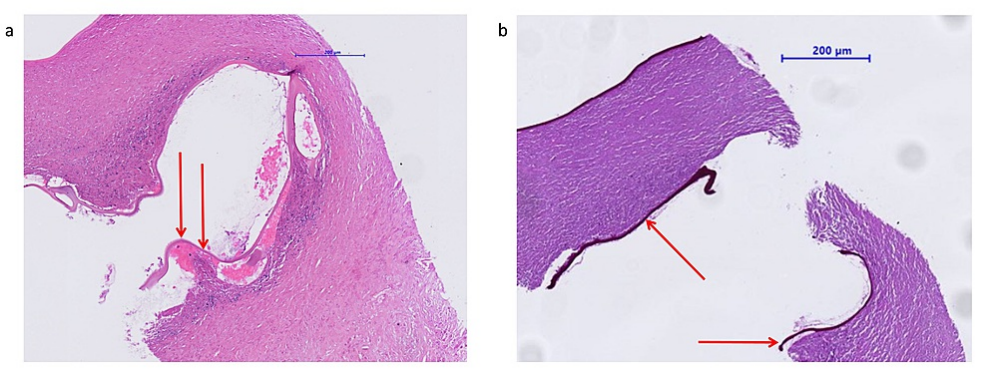
Liver biopsy showing alveolar echinococcosis The mass shows a multiloculated cyst with a germinal layer (arrows): (a) HE stain, (b) PAS stain. Abbreviations: HE = hematoxylin-eosin, PAS = periodic acid-Schiff

The CT scan of October 2020 showed new onset of multiple hypodense lesions of the liver, primarily suspected as metastases, compared to the CT of June 2020 (Figure 1). Therefore, a liver biopsy was performed, showing granulomatous demarcated necrosis zones and evidence of amorphous cuticular membranes with periodic acid-Schiff (PAS)-positivity compatible with *Echinococcus *spp*.* infestation (Figure 2). Malignancy could be excluded. The serology testing for *Echinococcus multilocularis* was positive (Table 1).

**Table 1:** Positive serology testing for Echinococcus multilocularis. Testing for *E. multilocularis* showed a positive Western blot but a negative result for *E. multilocularis* antigens (Em2 and Em18). An expression of an active AE is, in particular, a positivity of Em18, which, however, is not present here. The positivity for *E. granulosus* in the Western blot and EgHF ELISA is due to a cross-reaction with *E. multilocularis*. Abbreviations: AE = alveolar echinococcosis, EgHF = *E. granulosus* hydatid fluid antigen, ELISA = enzyme-linked immunosorbent assay

Name of investigation	Results	Normal ranges
*E. granulosus* EgHF, ELISA	22	< 1 AE
*E. multilocularis* Em2, ELISA	< 1	< 1 AE
*E. multilocularis* Em18, ELISA	< 1	< 1 AE
*E. granulosus*, Western Blot	weakly positive	negative
*E. multilocularis*, Western Blot	weakly positive	negative

Besides, liver enzymes and eosinophils were elevated (Table 2). A polymerase chain reaction (PCR) from the liver specimen confirmed *E. multilocularis* to be the infecting organism.

**Table 2:** Evolution of laboratory values Stage 1: Diagnosis of autoimmune encephalitis 04/2020. Stage 2: Under therapy with steroids (90mg/day). Stage 3: Diagnosis of AE 11/2020. Therapy with steroids stopped 1.5 months ago. Stage 4: Under therapy with albendazole for one month. Stage 5: Under therapy with albendazole for four months. Abbreviations: AE = alveolar echinococcosis, ALAT = alanine aminotransferase, AP = alkaline phosphatase, ASAT = aspartate aminotransferase, CRP = C-reactive protein, gamma-GT = gamma-glutamyltransferase

Name of investigation	Stage 1	Stage 2	Stage 3	Stage 4	Stage 5	Normal ranges
Leucocytes	5.88	8.07	7.28	6.0	6.66	3.0-10.5 G/L
Eosinophils	-	0.06	0.92	0.64	-	0.02-0.40 G/L
ASAT	24	38	70	57	40	< 50 U/L
ALAT	19	43	110	73	43	< 50 U/L
Bilirubin total	-	-	4	4	4	< 17 umol/l
AP	-	-	118	104	90	40-129 U/L
Gamma-GT	45	51	96	167	94	< 60 U/L
CRP	8	<3	12	6	4	< 5 mg/l

There was no involvement of other organs except the before-mentioned liver involvement. Continuous treatment with albendazole (400mg twice a day) was immediately initiated. The six-month follow-up showed no deterioration of the liver lesions in the CT scan, but liver enzymes normalized in the meantime.

## Discussion

This report describes a case of AE with disseminated liver involvement after initiation of immunosuppressive treatment with steroids and immunoglobulins due to a Ma-2 antibody-associated limbic encephalitis. In the literature, we do not find any correlation between Ma-2 antibody-associated limbic encephalitis and AE. In contrast, the role of immunosuppression in the development of AE is well known [5,6].

AE is a parasitic infection with a tumor-like and potentially invasive and metastasizing growth pattern. The current incidence of AE is three-fold higher than 20 years ago. This is associated with dense fox populations and high prevalence for *E. multilocularis*, respective urbanization of foxes, translocation of infected dogs, an increased frequency of medicating with immunosuppressive drugs, and modern imaging techniques that contribute to higher detection rates [2,3].

In immunocompetent individuals, infection with *E. multilocularis* may be controlled by the immune system, leading either to abortion of the parasite lesion or to a very slow-growing potential for the parasite, leading to late clinical manifestation [8]. Conversely, Lachenmayer et al. observed a higher incidence of AE in patients with immunocompromised conditions [5]. These results are comparable with data from a large French registry, which showed that almost 10% of the patients with AE had some immunosuppressive condition, including different malignancies, autoimmune diseases, or the intake of immunosuppressive drugs [4]. However, it is important to consider that type and extent of immune deficiency are difficult to compare between the different series.

In the study of Lachenmayer et al., there is no correlation between immunocompromised conditions and disease course [5]. In contrast, previous studies showed a rapid disease progression of AE in immunocompromised patients compared with immunocompetent patients [4,6].

Gottstein et al. showed three different disease courses of AE in general, depending on immune response elicited by the host: (i) seroconversion and the parasite fails to establish chronic infection, and detection of either no lesions, or only “dying” or “aborted” lesions; (ii) seroconversion and metacestodes grow slowly and establish a chronic infection, and first clinical symptoms occur putatively after five to 15 years post-infection, and (iii) uncontrolled and rapid metacestode proliferation, as it occurs in individuals with impaired immunity such as acquired immune deficiency syndrome (AIDS) patients or patients undergoing transplantation or being treated by immunosuppressive drugs or biological agents [8].

Hübner et al. discussed the T-cell-mediated immune response and showed that Th2-predominated immunity is associated with an increased susceptibility to disease in patients suffering from chronic AE. Conversely, protective immunity is induced by a Th-1-based immune response, even in aborted forms of AE [9]. A mix of a Th1/Th2 pattern in the chronic stages of AE is seen in most AE cases investigated [9].

Based on this knowledge, we can better understand the course of the disease in our patient. In fact, a therapy with glucocorticoids leads to an inhibition of the acute generation of both Th1- and Th2-derived cytokines by activated T cells, although the inhibitory effect on the expression of Th1 cytokines appears to be greater [10]. Thus, treatment with glucocorticoids may be associated with a shift in the expression of Th2-derived cytokines relative to Th1 cytokines [11]. In the case of infection with *E. multilocularis*, this imbalance favors an uncontrolled and rapid metacestode proliferation and thus explains the accelerated disease course of our patient.

In the case of our patient, steroids not only promoted the disease but also falsified the diagnosis. The negative serologies for Em2 and Em18 are probably due to the effect of steroids on antibody production. Corticosteroids used in higher than physiologic doses can also reduce the immune response to vaccines or other infections [12].

All these data raise the question of whether a screening program for AE before planned immunosuppressive therapy is useful and what such a program should include (e.g., serology, sonography). In case of the appearance of new liver lesions such as cysts or single or multiple tumor-like lesions, AE should always be included as a differential diagnosis, and further workup with an appropriate serology should be initiated [13].

The therapy of choice and the only curative approach available for AE is surgical resection. In unresectable cases, treatment with antihelminthic benzimidazoles (e.g., mebendazole, albendazole) has a markedly prolonged survival. Finally, intensified research on the treatment of this deadly disease is still necessary, especially to find parasiticidal compounds and supportive immunostimulatory medications.

## Conclusions

In conclusion, the increasing incidence of AE plays an important role in the management of patients, especially in the context of immunosuppressive therapy. In patients with immunosuppressive therapy, clinical presentation and diagnostics such as serology may be atypical or false-negative, respectively. In case of new findings or complaints after initiation of immunosuppressive therapy, AE should be sought at a low threshold.

Therefore, it is important that adequate screening for AE is considered for patients on immunosuppressive therapy, taking into account any influencing factors of immunosuppression on outcomes, e.g., false-negative results of serology. Furthermore, under established immunosuppressive therapy, close monitoring is indicated in order to recognise new complaints or findings at an early stage and to be able to adapt the further procedure accordingly.
